# Transverse Ridge Expansion and the Role of Bone Grafting: A Systematic Review

**DOI:** 10.7759/cureus.96558

**Published:** 2025-11-11

**Authors:** Salma Abdallaoui Maane, Driss Benazza

**Affiliations:** 1 Oral Surgery, Universidad Centro de Estudios Universitarios (CEU) San Pablo, Madrid, ESP; 2 Periodontology, International University of Rabat, Rabat, MAR

**Keywords:** alveolar ridge width, bone grafting, bone resorption, implant stability, transverse ridge expansion

## Abstract

This systematic review aimed to evaluate the role of bone grafting in transverse ridge expansion (TRE) by comparing clinical outcomes of procedures performed with and without grafting. An electronic search was conducted in PubMed, Embase, and the Cochrane Library. Fifteen clinical studies met the inclusion criteria, comprising randomized controlled trials (RCTs), cohort studies, and case series. The outcomes analyzed included implant survival, horizontal bone gain, marginal bone loss, and complications.

The findings revealed that both grafted and non-grafted TRE techniques achieved predictable ridge widening ranging from 3.2 to 6.1 mm, with implant survival rates consistently exceeding 92%. Although grafted sites showed slightly greater horizontal gains and improved ridge stability, these differences were modest and not clinically significant. The overall methodological quality of the included studies was moderate to low, with considerable heterogeneity in surgical techniques, grafting materials, and follow-up durations.

Within the limitations of the available evidence, TRE alone appears to be a reliable approach for ridges measuring ≥3 mm, whereas grafting may be indicated for narrower ridges or in cases requiring long-term volume preservation.

## Introduction and background

In contemporary dental implantology, the presence of adequate alveolar bone volume is a fundamental prerequisite for predictable implant placement and long-term functional stability. However, tooth loss, periodontal disease, trauma, and congenital factors often lead to horizontal bone resorption, resulting in narrow alveolar ridges that complicate or even preclude conventional implant insertion [1].

To overcome these limitations, several augmentation strategies have been proposed, including guided bone regeneration, block grafting, and alveolar distraction osteogenesis. Among these, transverse ridge expansion (TRE), first described by Scipioni et al. [2], has emerged as a conservative and minimally invasive technique designed to increase alveolar crest width without resorting to extensive grafting procedures. In parallel, other atraumatic surgical approaches, such as the osteotome sinus floor elevation introduced by Summers [3], have also been developed to optimize implant placement in compromised anatomy.

The TRE technique involves a controlled separation of the buccal and lingual/palatal cortical plates, thereby creating a biological space that can either be left to heal spontaneously or filled with grafting materials, depending on the clinical situation. When performed correctly, TRE can result in horizontal bone gains ranging from 4 to 6 mm [4,5], often sufficient to allow implant placement in ridges initially measuring as little as 3 mm [1]. Nonetheless, the role of adjunctive bone grafting in this context remains a subject of debate. While some clinicians recommend grafting in nearly all expansion cases to promote stability and osteogenesis [6,7], others argue that, in ridges measuring ≥3 mm, expansion alone can provide predictable outcomes without additional grafting [2,3].

Variability in surgical technique [8,9], patient-specific bone quality, and differences in biomaterials [10,11] further complicate the establishment of standardized protocols. Moreover, the introduction of piezosurgery [12], digital planning [13], and biologically active adjuncts such as plasma rich in growth factors (PRGF) [14] have refined surgical performance and may influence the necessity of bone grafting.

Search parameters

The present systematic review was conducted according to the Preferred Reporting Items for Systematic Reviews and Meta-Analyses (PRISMA) guidelines. Electronic searches were performed in PubMed, Embase, and the Cochrane Library for studies published between January 2000 and December 2024, limited to English-language human clinical studies.

PICO framework

Population

We included patients presenting with horizontally deficient alveolar ridges requiring implant placement.

Intervention

The intervention consisted of transverse ridge expansion (TRE) with adjunctive bone grafting.

Comparison

The comparison group underwent transverse ridge expansion without bone grafting.

Outcomes

The outcomes assessed were horizontal bone gain, implant stability, survival rate, marginal bone loss, and complication incidence.

Given these discrepancies, a systematic appraisal of the available evidence is warranted. Therefore, the objective of this systematic review is to critically assess whether bone grafting confers a significant advantage in transverse ridge expansion procedures, synthesizing data from clinical studies to provide evidence-based guidance for implant treatment planning and clinical decision-making.

Study objectives

The primary objective was to evaluate whether the use of bone grafting during transverse ridge expansion (TRE) improves clinical outcomes compared with TRE performed without grafting. The secondary objectives were to compare implant stability, survival rates, marginal bone resorption, and the incidence of complications between the two approaches.

## Review

Materials and methods

Study Design

The review followed the Preferred Reporting Items for Systematic Reviews and Meta-Analyses (PRISMA) 2020 reporting guidelines, and the protocol was prospectively registered in PROSPERO under registration number CRD420251158137, ensuring methodological transparency, reproducibility, and adherence to systematic review standards.

Search Strategy

The electronic search was conducted in PubMed, Embase, and the Cochrane Library using the following keywords and Boolean operators: (“ridge expansion” OR “split-crest” OR “alveolar expansion”) AND (“dental implant” OR “implant placement”) AND (“bone graft” OR “bone regeneration”). The search was limited to human studies published in English between January 2000 and December 2024 (database versions: PubMed (NCBI platform), Embase (Elsevier), and Cochrane Library (Wiley)).

Selection Process

Two independent reviewers screened all records at the title/abstract and full-text levels. Discrepancies were resolved by consensus. The inter-reviewer agreement was high (Cohen’s kappa = 0.86), indicating excellent reliability in the selection process.

Inclusion criteria: Human clinical studies (randomized controlled trials (RCTs), prospective or retrospective cohort studies, and case series with ≥10 patients), studies evaluating TRE with or without bone grafting, and studies with a minimum follow-up period of six months were included.

Exclusion criteria: We excluded animal or in vitro studies, case reports including fewer than 10 patients, narrative reviews, expert opinions, and studies lacking clinical or radiographic outcome data.

Data Extraction

For each included study, the following data were extracted and tabulated: study design and sample size, baseline ridge width and dimensions, surgical technique and grafting protocol (if applicable), follow-up duration, implant survival and marginal bone loss, and reported complications. Data extraction was performed independently by two reviewers to ensure accuracy and consistency.

Risk of Bias Assessment

The methodological quality of the included studies was assessed according to their design. Randomized controlled trials (RCTs) were evaluated using the Cochrane Risk of Bias 2 (RoB-2) tool [15], and non-randomized studies (cohort and case series) were assessed using the Risk of Bias In Non-randomised Studies of Interventions (ROBINS-I) tool [16].

The following domains were considered: selection bias, performance bias, detection bias, attrition bias, reporting bias, and publication bias. Each study was categorized as having low, moderate, or high risk of bias. All assessments were performed independently by two reviewers, and discrepancies were resolved by consensus.

Results

Study Selection and Characteristics

**Figure 1:**
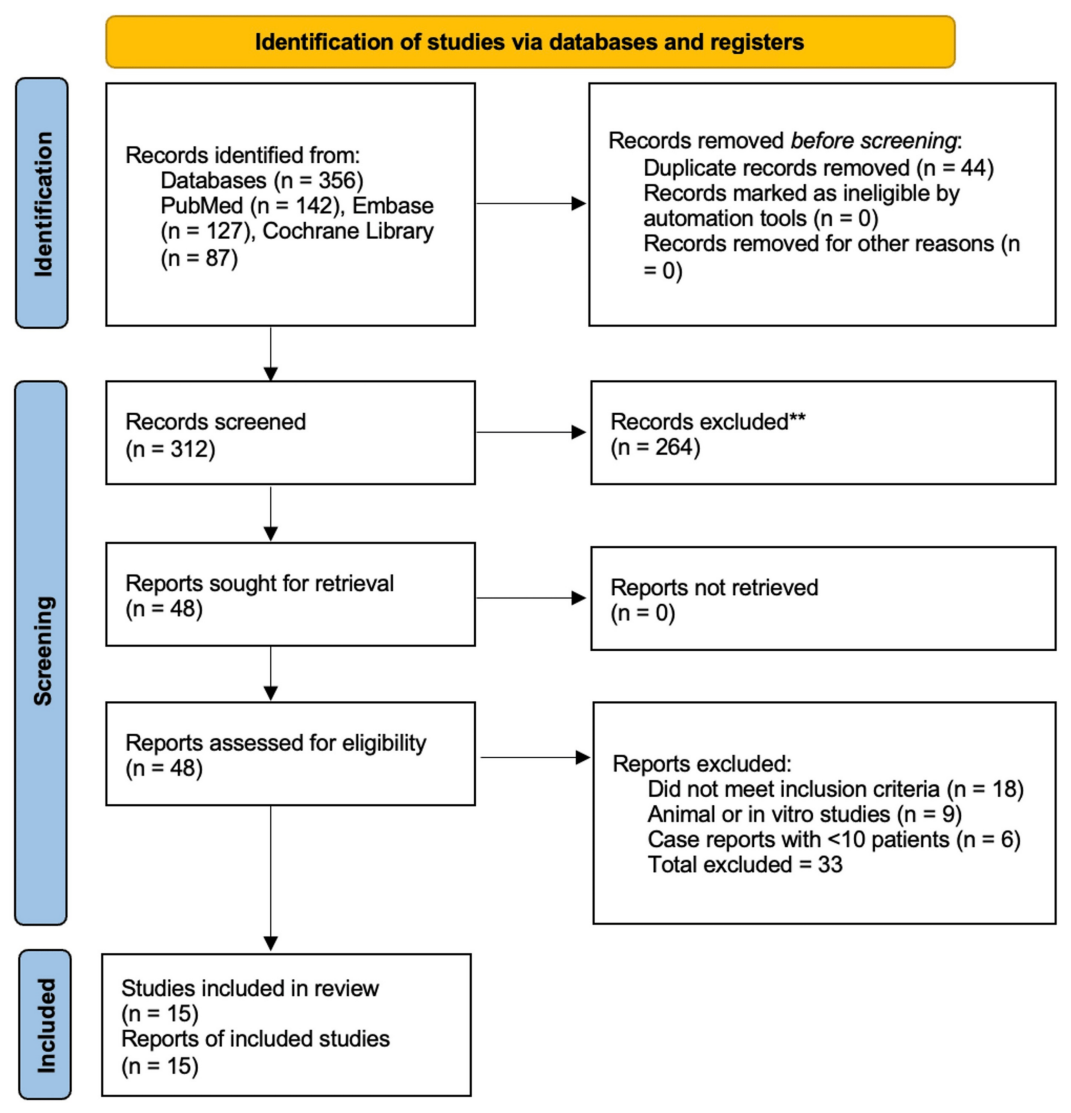
PRISMA 2020 flow diagram illustrating the study identification, screening, eligibility assessment, and inclusion process PRISMA: Preferred Reporting Items for Systematic Reviews and Meta-Analyses

A total of 356 records were identified through PubMed, Embase, and the Cochrane Library. After removing duplicates and screening titles and abstracts, 48 full-text articles were assessed for eligibility. Finally, 15 studies met the inclusion criteria and were analyzed. The study selection process is summarized in the PRISMA 2020 flow diagram (Figure 1).

The included studies comprised five randomized controlled trials (RCTs), six prospective cohort studies, and four retrospective case series, with follow-up durations ranging from six months to five years. Baseline ridge widths varied between 2.5 and 4.5 mm, and sample sizes ranged from 20 to 120 patients. A summary of the included studies is presented in Table 1.

**Table 1:** Characteristics and outcomes of the 15 clinical studies included in this systematic review, evaluating transverse ridge expansion with or without bone grafting Data include study design, sample size, baseline ridge width, technique, grafting protocol, horizontal bone gain, and follow-up. GTR: guided tissue regeneration

Author (year)	Type of study	Level of evidence	Baseline ridge width (mm)	Technique	Graft used	Mean horizontal bone gain
Simion et al. (1992) [1]	Prospective case series	Level 4	3	Split-crest + GTR	Membrane	1-4 mm
Scipioni et al. (1994) [2]	Prospective case series	Level 4	3	Transversal expansion	None	3-4 mm
Ferrigno and Laureti (2005) [4]	Retrospective cohort	Level 3	≈3-4	Split-crest	None	3 mm
Bravi et al. (2007) [14]	Case series	Level 4	4	Transversal expansion	None	2-3 mm
Blus and Szmukler-Moncler (2010) [10]	Prospective cohort	Level 3	3.25	Split-crest (ultrasonic bone surgery)	None	3.5 mm
Sethi and Kaus (2000/2013) [11]	Case series	Level 4	2-4	Maxillary ridge expansion	Closed healing (± graft)	3-6 mm
Nkenke et al. (2018) [17]	Prospective cohort	Level 3	2.5-3.0	Split-crest/piezo	Autogenous block	≈6.1 mm
Blus et al. (2013) [18]	Prospective cohort	Level 3	≈3-4	Split-crest (piezo)	Autograft + Bio-Oss	5-6.5 mm
González-García et al. (2011) [19]	Prospective cohort	Level 3	3-4	Modified split-crest osteotomy	Autograft	4-5 mm
Holtzclaw et al. (2010) [20]	Case series	Level 4	Posterior mandible	Piezo hinge-assisted ridge split	None	2 mm
Altiparmak et al. (2017) [21]	Randomized controlled trial	Level 2	Not reported	Ridge split versus autogenous onlay bone graft	Split ± graft	±5 mm
Rahpeyma et al. (2013) [22]	Case series	Level 4	Not reported	Lateral ridge split + immediate implants	None	2-3 mm
Crespi et al. (2021) [23]	Randomized controlled trial	Level 2	Atrophic arches (not reported)	Split-crest (maxilla and mandible)	±Grafts	3-5 mm
Manekar et al. (2022) [24]	Case series	Level 4	1.5-3.0 (mandible)	Alveolar ridge split and expansion	None	1-1.5 mm
Enislidis et al. (2006) [25]	Prospective cohort	Level 3	≈3	Minimally invasive lateral ridge splitting	None	2-4 mm

Bone Gain and Implant Stability

All studies reported significant ridge width increases following TRE, with horizontal bone gains ranging from 3.2 to 6.1 mm. When bone grafting was performed using autogenous, xenogeneic, or alloplastic materials, the mean gain was slightly higher (4.5-6.1 mm) than in non-grafted sites (3.2-5.0 mm). Implant primary stability, assessed by insertion torque or implant stability quotient (ISQ), tended to be greater in grafted groups, although differences were not statistically significant in most studies.

Implant Survival and Success Rates

Across all studies, implant survival ranged from 92% to 100%, with no significant difference between grafted and non-grafted groups. Implant success, defined as marginal bone loss < 1.5 mm, absence of mobility, and no peri-implant infection, was also comparable between techniques, confirming the predictability of both approaches.

Complications

The most common complications were buccal plate fractures (4%-10%), soft tissue dehiscence (3%-8%), and occasional autogenous graft resorption. Minor horizontal relapse (<1 mm) was sometimes observed in non-grafted sites, whereas grafted ridges showed better volume stability. However, overall complication rates remained similar between treatment modalities.

Influence of Biomaterials and Techniques

Among grafted sites, xenografts (e.g., Bio-Oss®) and alloplastic substitutes demonstrated better long-term dimensional stability than autogenous bone, which was more prone to partial resorption. The use of piezosurgery instead of rotary instruments consistently reduced complication rates and improved expansion control. Recent studies also explored digital planning and biologic enhancers, such as plasma rich in growth factors (PRGF), although current evidence remains heterogeneous and limited in scope.

Overall Evidence Summary

Taken together, the findings indicate that TRE alone achieves predictable results when the initial ridge width is ≥3 mm, while bone grafting may be advantageous in narrower ridges or when long-term stability is desired. Comprehensive data supporting these observations are provided in Table 1.

Risk of Bias Assessment

**Figure 2:**
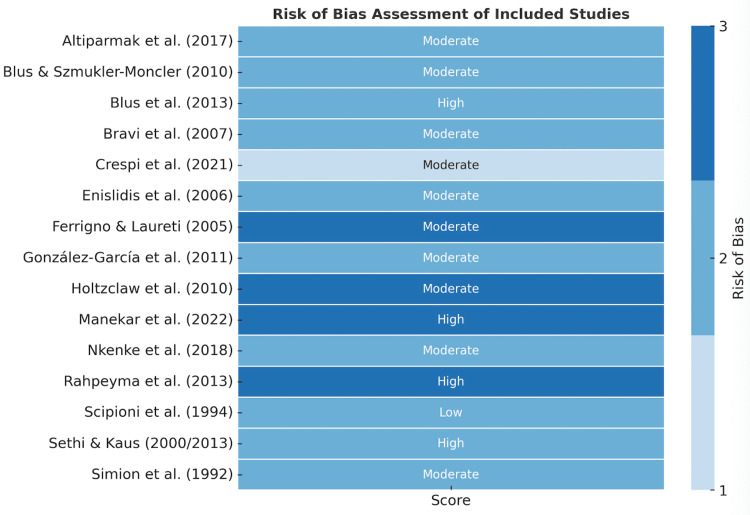
Risk of bias assessment of the included studies

Of the 15 included studies, two were RCTs (one low risk and one moderate), six were prospective cohorts (mostly moderate risk), and seven were case series (moderate to high risk). The main bias sources were patient selection, variability in surgical technique, lack of assessor blinding, and incomplete reporting of complications. Most studies featured small sample sizes and short- to mid-term follow-up, limiting the strength of the evidence. A summary of risk of bias evaluations is presented in Figure 2.

Discussion

The findings of this systematic review provide valuable insights into the role of bone grafting in TRE. Across the 15 included studies, horizontal bone gain ranged from 3.2 to 6.1 mm, confirming that TRE is an effective and predictable technique for widening narrow alveolar ridges and enabling successful implant placement [24].

Bone Gain and Stability

Several studies demonstrated significant ridge widening following TRE without adjunctive grafting. Blus and Szmukler-Moncler reported an increase from 3.25 to 6.5 mm across 230 sites [10], while Bravi et al. observed consistent gains of 2-5 mm in a large multicenter cohort [14]. Similarly, Waechter et al. conducted a systematic review and meta-analysis reporting an average horizontal gain of 3.8 mm across 732 patients, confirming the efficacy of TRE in achieving predictable ridge expansion [26].

When bone grafting was used, mean horizontal gains were slightly higher. Nkenke et al. reported 6.1 mm with autogenous block grafts [17], while Urban et al. achieved 5.8 mm using a combination of autografts and xenografts [27]. Studies by Altiparmak et al. [21] and Crespi et al. [23] also confirmed greater dimensional stability when graft materials were used. Nevertheless, although these differences were numerically greater, their clinical relevance remains limited, as implant survival rates were comparable between grafted and non-grafted groups [11,14,22-27].

TRE also appeared more effective in the maxilla than in the mandible due to its lower cortical density and higher bone elasticity [17,19,23,25]. This may explain the higher predictability and lower incidence of cortical plate fractures observed in maxillary compared with mandibular sites.

Although grafted sites demonstrated slightly greater horizontal bone gains than non-grafted ones, these differences were modest and did not translate into clinically significant improvements. Implant survival rates remained consistently high across all studies, regardless of grafting.

Implant Survival and Long-Term Outcomes

Implant survival rates consistently ranged from 92% to 100%, irrespective of grafting [2,10,11,14,18-24]. González-García et al. demonstrated stable ridge width and long-term success (>2 years) in crests initially 3-4 mm wide [19]. These outcomes suggest that baseline ridge width and surgical technique exert a greater influence on implant prognosis than grafting itself [5,17,21].

Complications and Risk Factors

Reported complications were generally minor. Buccal plate fractures (4%-10%) and soft tissue dehiscence (3%-8%) were the most frequent [4,6,9]. Autogenous grafts showed partial resorption, whereas xenografts and alloplasts demonstrated superior stability [7,19,23]. Non-grafted cases occasionally presented minor horizontal relapse (<1 mm), but this did not compromise implant survival [10,11,19].

Influence of Technique and Adjunctive Technologies

Differences in outcomes may be linked to variations in surgical approach and the use of adjunctive technologies. Piezosurgery was consistently associated with lower complication rates and more controlled expansion compared with traditional burs or chisels [10,21,28].

Recent regenerative strategies, including the use of platelet concentrates such as platelet-rich plasma (PRP) and injectable platelet-rich fibrin (i-PRF), have shown promising results in enhancing bone regeneration and soft tissue healing. Idris et al. highlighted in a recent systematic review the regenerative potential of i-PRF in promoting bone maturation and implant integration [29,30]. Similarly, Ammar et al. demonstrated in a randomized clinical trial that i-PRF led to superior early healing and bone quality compared to PRP, supporting its role as an adjunctive biologic enhancer in implant-related regenerative procedures [31].

Emerging techniques such as digital planning [12,13] and biologically active agents such as PRGF [28] may enhance healing, bone formation, and workflow reproducibility, as supported by Jaber et al. [32], who emphasized the accuracy and clinical reliability of digital implantology workflows. However, the current evidence remains limited and heterogeneous.

Clinical Implications

Current evidence supports that TRE alone provides safe and predictable outcomes for ridges measuring ≥3 mm, eliminating the need for routine grafting [1,2,10,14,16,22]. However, bone grafting remains indicated for ridges < 3 mm or in cases requiring additional ridge stability and long-term volume preservation [17,19,21,23].

Overall, while the addition of bone grafting may yield slightly greater horizontal bone gain, this advantage appears modest and of limited clinical significance. Implant survival and success remain predictably high, irrespective of grafting. The adjunctive use of biologic enhancers such as PRP or i-PRF may further improve regenerative outcomes, but high-quality clinical evidence is still required to validate their long-term benefits in TRE.

Table 2 provides a summary of heterogeneity among the included studies.

**Table 2:** Summary of heterogeneity among the included studies Summary of heterogeneity among the 15 included studies regarding initial ridge width, grafting material, surgical approach, and follow-up duration. Variations in these parameters limited comparability and prevented quantitative synthesis. GTR: guided tissue regeneration

Author (year)	Initial ridge width (mm)	Grafting material	Surgical technique	Follow-up duration	Main source of heterogeneity
Simion et al. (1992) [1]	3	Membrane, GTR	Split-crest	12 months	Graft type, limited sample
Scipioni et al. (1994) [2]	3	None	Transversal expansion	60 months	Technique, long follow-up
Ferrigno and Laureti (2005) [4]	3-4	None	Split-crest	24 months	Technique variation
Bravi et al. (2007) [14]	4	None	Transcrestal expansion	24 months	Technique, patient variability
Blus and Szmukler-Moncler (2010) [10]	3.25	None	Ultrasonic split-crest	36 months	Technique (piezosurgery)
Sethi and Kaus (2013) [11]	2-4	±Graft	Ridge expansion	60 months	Mixed graft protocols
Nkenke et al. (2018) [17]	2.5-3	Autogenous block	Split-crest (piezo)	24 months	Graft material
Blus et al. (2013) [18]	3-4	Autograft + Bio-Oss®	Split-crest (piezo)	36 months	Technique and graft type
González-García et al. (2011) [19]	3-4	Autograft	Modified split-crest osteotomy	24 months	Technique variation
Holtzclaw et al. (2010) [20]	Posterior mandible	None	Piezo hinge-assisted ridge split	12 months	Anatomic site
Altiparmak et al. (2017) [21]	NR	±Graft	Ridge split versus onlay graft	24 months	Graft protocol
Rahpeyma et al. (2013) [22]	NR	None	Lateral ridge split + immediate implants	12 months	Technique
Crespi et al. (2021) [22]	Atrophic arches	±Graft	Split-crest (maxilla and mandible)	60 months	Follow-up duration
Manekar et al. (2022) [23]	1.5-3.0	None	Alveolar ridge split and expansion	36 months	Ridge width, site
Enislidis et al. (2006) [24]	≈3	None	Minimally invasive lateral split	12 months	Technique

Future Perspectives

Future research should focus on conducting randomized controlled trials with standardized surgical protocols, uniform outcome measures, and long-term follow-up to enhance comparability across studies. Comparative trials evaluating grafted versus non-grafted TRE within specific ridge width categories are needed to define clinical thresholds more precisely. Moreover, the integration of piezosurgery, digital planning, and biologic adjuncts such as PRGF represents a promising avenue for optimizing bone regeneration and improving treatment predictability.

## Conclusions

This systematic review confirms that transverse ridge expansion (TRE) is a conservative, predictable, and effective technique for managing narrow alveolar ridges. Reported horizontal bone gains ranged from 3.2 to 6.1 mm, with implant survival consistently high (92%-100%). In ridges measuring ≥3 mm, expansion alone generally provides sufficient width for implant placement, whereas bone grafting appears most beneficial in cases of severe deficiency (<3 mm) or when additional dimensional stability and long-term volume preservation are required. Complication rates, such as buccal plate fractures and soft tissue dehiscence, remain low and manageable. Although grafted sites demonstrated slightly greater horizontal gains, these differences were modest and did not translate into clinically significant improvements, reinforcing that grafting should be considered a selective strategy rather than a routine adjunct.

Emerging technologies, including piezosurgery, digital planning, and biologically active adjuncts such as PRGF, may further enhance treatment predictability and patient outcomes. However, stronger evidence from well-designed randomized controlled trials with standardized protocols, uniform outcome measures, and long-term follow-up is needed to establish consistent, evidence-based clinical guidelines. In summary, TRE represents a minimally invasive and reliable approach for horizontal ridge augmentation, while bone grafting should remain a case-dependent option reserved for specific clinical indications.
